# Urban Power Line Corridors as Novel Habitats for Grassland and Alien Plant Species in South-Western Finland

**DOI:** 10.1371/journal.pone.0142236

**Published:** 2015-11-13

**Authors:** Jussi Lampinen, Kalle Ruokolainen, Ari-Pekka Huhta

**Affiliations:** Department of Biology, University of Turku, Turku, Finland; University of Sydney, AUSTRALIA

## Abstract

Regularly managed electric power line corridors may provide habitats for both early-successional grassland plant species and disturbance-dependent alien plant species. These habitats are especially important in urban areas, where they can help conserve native grassland species and communities in urban greenspace. However, they can also provide further footholds for potentially invasive alien species that already characterize urban areas. In order to implement power line corridors into urban conservation, it is important to understand which environmental conditions in the corridors favor grassland species and which alien species. Likewise it is important to know whether similar environmental factors in the corridors control the species composition of the two groups. We conducted a vegetation study in a 43 kilometer long urban power line corridor network in south-western Finland, and used generalized linear models and distance-based redundancy analysis to determine which environmental factors best predict the occurrence and composition of grassland and alien plant species in the corridors. The results imply that old corridors on dry soils and steep slopes characterized by a history as open areas and pastures are especially suitable for grassland species. Corridors suitable for alien species, in turn, are characterized by productive soils and abundant light and are surrounded by a dense urban fabric. Factors controlling species composition in the two groups are somewhat correlated, with the most important factors including light abundance, soil moisture, soil calcium concentration and soil productivity. The results have implications for grassland conservation and invasive alien species control in urban areas.

## Introduction

To ensure the secure transport and distribution of electricity, the vegetation in power line corridors is maintained low with regular management, such as mechanical clear cuts or herbicide sprays [1, 2]. Ecologically this management acts as frequent disturbance that keeps the corridor vegetation in an early stage of succession. In terms of environmental conditions, the removal of tall-growing species enhances temperature fluctuations and light intensity near the ground, increases evaporation and decreases nutrient uptake from the soil [1, 2, 3].

Because of their management, power line corridors may provide novel habitats for early-successional plant species [4, 5, 6, 7, 3]. This is especially important for species adapted to semi-natural grasslands, habitats created by traditional agriculture. Many of those species have become endangered throughout Europe because of land-use changes during the 20^th^ century and the resulting decline in grassland area [8, 9]. At present the species of traditional rural biotopes, including semi-natural grasslands, comprise the second largest groups of red-listed species in both Finland and Sweden [10, 11]. In addition, the proportion of endangered or critically endangered habitat types in Finland is highest in traditional rural biotopes [12].

On the other hand, power line corridors may also provide suitable habitats for alien species [13, 6] and facilitate their dispersal [14, 15]. This is because alien species tend to occupy disturbed habitats often characterized by a surplus of unused resources [16, 17], both of which are typical features for regularly managed power line corridors [2, 13]. The spread of alien species along power line corridors may increase the likelihood of their invasion to the surrounding habitats, potentially decreasing native species diversity, altering ecosystem processes or causing other adverse effects related to invasion [18].

As power line corridors have implications for both the conservation of grassland species and the spread of alien species, it is important to understand which environmental factors control the occurrence and composition of the two species groups in the corridors. This would help managers to plan corridor management to favor grassland species in the types of corridors best suited for them, and to deter the spread of alien species in the types of corridors most susceptible to invasion. Such planning enables power line corridors to be used in cities to promote native grassland vegetation, and to prevent the further spread of alien species, thus counteracting the biotic homogenization typical to urban areas [19, 20].

This study aims to (1) identify the environmental factors that best predict the occurrence of grassland and alien plant species in urban power line corridors and (2) determine to what extent the environmental factors affecting the species composition in the two groups are similar. Based on previous knowledge on grassland [21, 22] and invasion ecology [16, 20], we predict that the greatest numbers of grassland species will be found in corridors with abundant light, while the greatest numbers of alien species will be found in corridors that are close to urban areas and have been recently managed.

## Materials and Methods

### Study area

**Fig 1:**
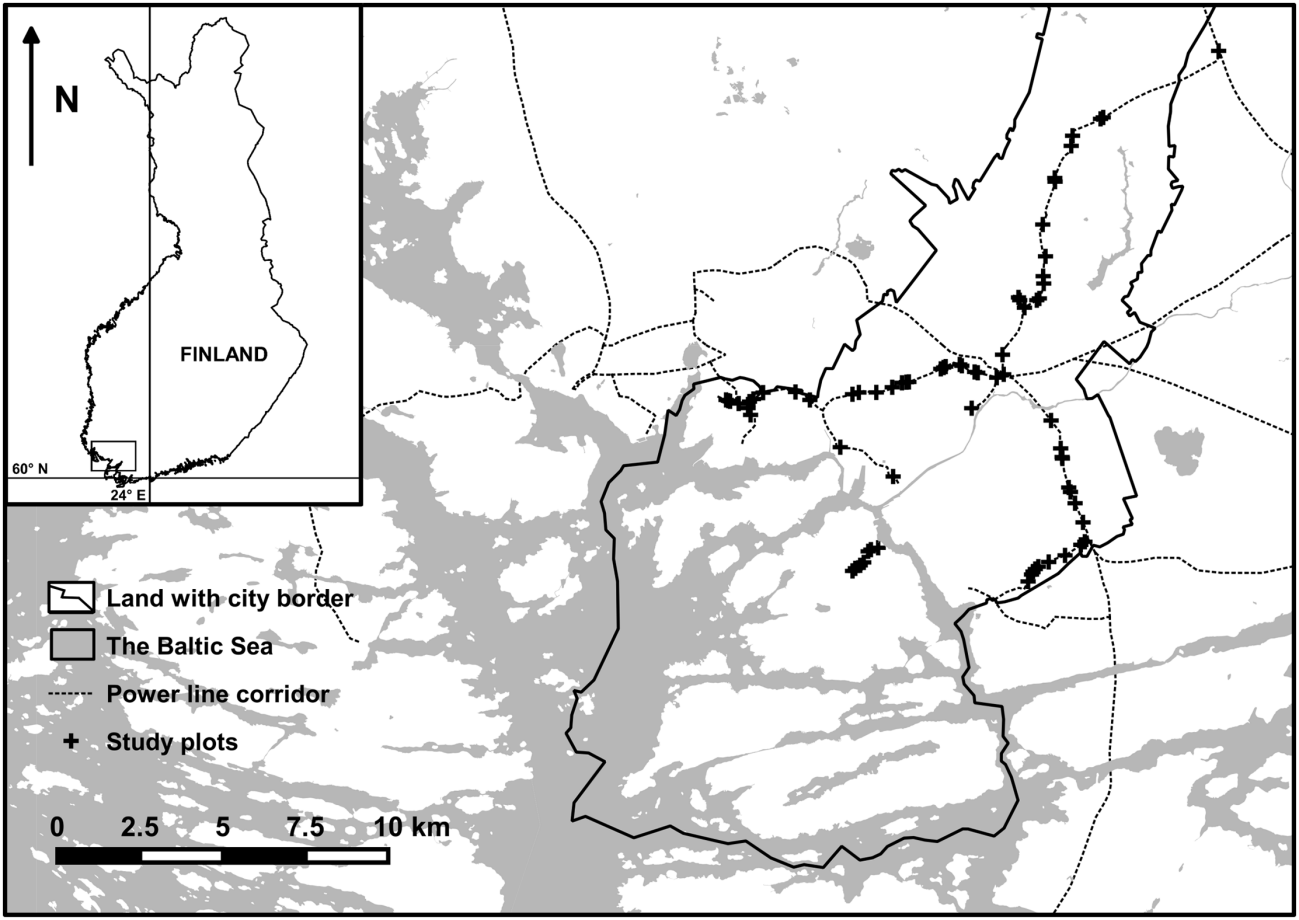
Map of the study plots in electric power line corridors in Turku, south-western Finland. The map is based on open access cartographic material [24] and the coordinates of the study plot, compiled with QGIS version Chugiak 2.4.0 [25].

The study area is located in south-western Finland, on the northern edge of the hemiboreal vegetation zone [23]. The study was conducted in a 43 kilometer long and approximately 26 meter wide 110 kV power line corridor network in the city of Turku (Fig 1). The corridors cross various habitats surrounding the city center, such as cultivated or abandoned fields on clay soil, sub-xeric heath forests on shallow soils, urban roads and suburban areas. The land in the corridors is owned mostly by the city, but managed by the local electric company. Corridor management follows a 3-year cycle, so that each year one third of the network is cut clear of any vegetation higher than 3 meters. The produced woody debris is mostly left on the corridor floor.

### Field work

#### Sampling

The field work of the study was conducted as a part of a vegetation survey in the power line corridor network ordered by the local electric company Turku Energia Oy. The objective of the survey was to chart the vascular plants and vegetation types present in corridors crossing different habitat types. For this reason, the studied corridors were first divided into five major habitat types: urban/suburban areas, densely forested areas, agricultural areas, wetland areas and areas with rocky outcrops. Plot locations for the study were chosen from these, based on preliminary field observations of either grassland or alien species. Areas with no signs of management or present vegetation (parking lots, highways etc.) were not included in the sampling. The average distance between plots was 326.7 meters (min. 44.6 m and max. 4029.5 m).

As the sampling scheme includes subjectivity, the final set of plots does not constitute a random sample of environmental conditions on the corridors. The sampling cannot thus be used to draw generalizations on the environmental conditions on the studied corridors. However, the subjectivity does not affect a comparison between the environmental conditions the encountered grassland and alien species occur in.

#### Species data

Species abundance data on vascular plants was gathered using 71 plots measuring 25 x 25 meters between May 21^st^ and July 10^th^ 2012. Plot size was chosen according to the prevalent corridor width in the study area (26 meters). The species on the plot were identified using national floras [26, 27], and their coverage on the plot was estimated with an ordinal scale ranging from 1 to 9 (adapted from [28]).

When applicable, the encountered species were classified as either grassland species or alien species, according to previous studies on grassland ecology [29], national floras [26, 27] and Finland’s National Strategy on Invasive Alien Species [30]. Both species groups were further divided in two: Grassland species into common species and indicator species, and alien species into indifferent and invasive species [29, 30]. Common grassland species and grassland indicator species are both associated with grasslands created by traditional agriculture. However, the first are less specialized according to their habitat preferences, and often occur also in other types of habitats, such as road verges and lawns. Grassland indicator species on the other hand are found only in the vicinity of long lasting traditional agriculture, and tend to disappear from grasslands as management ceases [21]. In turn, indifferent and invasive alien species both occur in the study area outside their native range and have been introduced there due to human action after the early 17^th^ century [26]. The first comprise non-invasive, often ephemeral species that have not caused severe impacts in native environments in Finland. The latter are regarded as either locally or nationally harmful invaders with potentially detrimental impacts on native species and communities [30].

#### Environmental data

Recorded environmental variables are listed in Table 1 (adapted from [7]). Data on corridor management was obtained from the electric company. Data on historical land cover types with 5 classes predating the power lines was recorded from digitized Russian topographic maps from the 1880’s [31], georeferenced with present day basic maps (1:20 000). To gain information on the present surrounding environment of each corridor, the percentage cover of four different present-day land cover types was recorded on a 100 m wide circle around each plot. The types were taken from CORINE 2012 land cover data, with a resolution of 20 x 20 meters [32]. As the distances between plots did not exceed 100 meters in all cases, 11 circles overlapped in part with another, causing the data on surrounding land cover to become redundant to a small extent. The historical and present land cover data was obtained with QGIS version Chugiak 2.4.0 [25].

**Table 1:** Plant species and environmental data used in the study, with abbreviations for individual variables.

Data	Description	Source
**Species data:**	Coverage data on encountered species, evaluated with 9 category values: **1** = < 0.125% coverage, **2** = 0.125–0.5%, **3** = 0.5–2%, **4** = 2–4%, **5** = 4–8%, **6** = 8–16%, **7** = 16–32%, **8** = 32–64% and **9** = > 64% coverage.	Field work
**Corridor physical properties and management:**		
Corridor age / Age	Age of corridor in years.	Electric company
Slope steepness / SlopeSt	Steepness expressed with 4 ordinal classes: **1** = Level ground, **2** = modest, **3** = steep and **4** = very steep slope.	Field work
Slope direction / SlopeDr	Direction expressed with 3 ordinal classes: **1** = North, **2** = East, West or level ground, **3** = South.	Field work
Corridor width / Width	Width expressed with 4 ordinal classes: **1** = < 26 meters, **2** = 26 m, **3** = 26–50 m and **4** = > 50 m.	Field work
Time since management / Time	Time in years since the previous clear-cut of the corridor.	Electric company
Amount of debris / Debris	Expressed with 5 ordinal classes: **1** = No debris, **2** = Small, **3** = Moderate, **4** = Large and **5** = Excessive amounts of debris.	Field work
Shrub density / ShrubD	Density of shrubs and saplings, expressed with 5 ordinal classes: **1** = No shrub layer, **2** = Sparse, **3** = Somewhat dense, **4** = Very dense and **5** = Extremely dense shrub layer.	Field work
**Ellenberg indicator values:**	Plot-wise mean indicator values based on the values of species found on each plot. The studied species in each analysis were omitted from the calculation in order to avoid circular reasoning.	[33, 34]
Ellenberg light abundance / ELight		
Ellenberg soil moisture / EMoisture		
Ellenberg soil calcium / ECalcium		
Ellenberg soil productivity / EProd		
**Present-day land cover:**	Cover of CORINE land cover classes on a circle with 100 m diameter surrounding the plot	[32]
Cover of surrounding urban fabric / CovUrb	Cover (%) of CORINE class 1 (levels 1–4),	
Cover of surrounding artificial surfaces / CovArt	Cover (%) of CORINE class 1 (levels 1–15),	
Cover of surrounding agricultural areas / CovAgr	Cover (%) of CORINE class 2 (levels 16–21) and	
Cover of surrounding forests and semi-natural areas / CovFor	Cover (%) of CORINE class 3, (levels 22–38, excluding 36)	
**Historical land cover:**	Categorical variable describing land cover on plot location in the late 1800´s. Corresponding present CORINE classes are expressed in brackets.	Topographical map (1880) [31]
History: Bedrock / H:Bed (class 3)	Outcrops of bare bedrock	
History: Open areas and pasture / H:Pas (class 2)	Non-forested, open areas unsuitable for cultivation and often used as pastures	
History: Grassland / H:Grass (class 2)	Mesic, often lowland areas used for hay-making and grazing	
History: Cultivated field / H:Cult (class 2)	Staple crops	
History: Forest / H:For (class 3)	Forested areas	
**Autocovariate / Autocov:**	A measure of spatial autocorrelation. Describes the effect of neighboring plots on the response variable.	Analyses [35]

Soil and light conditions on each plot were inferred with four Ellenberg -indicator values [33, 34], calculated as averages of the species-specific indicator values of the species found on the plot. Common grassland species, grassland indicator species and indifferent and invasive species were omitted from their respective analyses when calculating these values in order to avoid circular conclusions [S2 Table]. The indicator values for nitrogen and soil reaction have been shown to represent a broader measure of soil productivity and soil calcium concentration, respectively [36, 37]. These are as such referred to as productivity- and calcium values in this study.

### Numerical analyses

#### Generalized linear models

Generalized linear models were used to identify the environmental factors that best predict the number of common grassland species, grassland indicator species and indifferent and invasive alien species in the studied corridors. Poisson error distribution was chosen for the response variable, as it fits well with count-based data [38]. Before building the models, the categorical variable describing historical land cover was transformed into dummy-variables, and these and all other environmental variables were standardized. Four preliminary models were then built, with all environmental variables as predictors and the species counts of common and rare grassland species and indifferent and invasive alien species respectively as response variables. To account for spatial autocorrelation, an autocovariate was included in each model. The autocovariate was based on the inversely distance weighted effect of other plots on the response variable [39, 35]. The predictors in the models were examined for multicollinearity by comparing their variance inflation factors (VIF’s) in the preliminary models and by calculating pairwise Pearson correlations between them (S3 Table). Variables with a VIF -value greatly exceeding 10 in all analyses were omitted [40] with the exception of dummy-variables describing historical land cover. After this, the amount of predictor variables in the models was reduced with backward elimination based on model AIC-values. Finally, the *p*-values in the final models were corrected for false discovery rate [41].

#### Distance based redundancy analysis

Distance based redundancy analysis [42] was used to measure the amount of variation the collected environmental data could explain in the composition of both grassland and alien species, and to rank the individual variables according to the amount of explained variation. As only plots containing species from both groups of species could be selected for the analysis, the division into common grassland species and grassland indicator species and into indifferent and invasive alien species was collapsed into simply grassland and alien species (*n* = 67 plots). All environmental variables were standardized and then analyzed together with the species data (measured with Bray-Curtis distance), with significance tests of 500 permutations for each variable. This was replicated for both species groups, after which the variables were analyzed individually with the respective species data and ranked according to the amount of explained variation. Spearman correlation was then calculated for the amount of variation each variable could explain in the two species groups.

All numerical analyses were conducted with R version 3.1.2 and the associated packages: stats [43], car [44], spdep [45] for generalized linear models and ecodist [46] and vegan [47] for distance based redundancy analyses.

## Results

### Species occurrences

**Fig 2:**
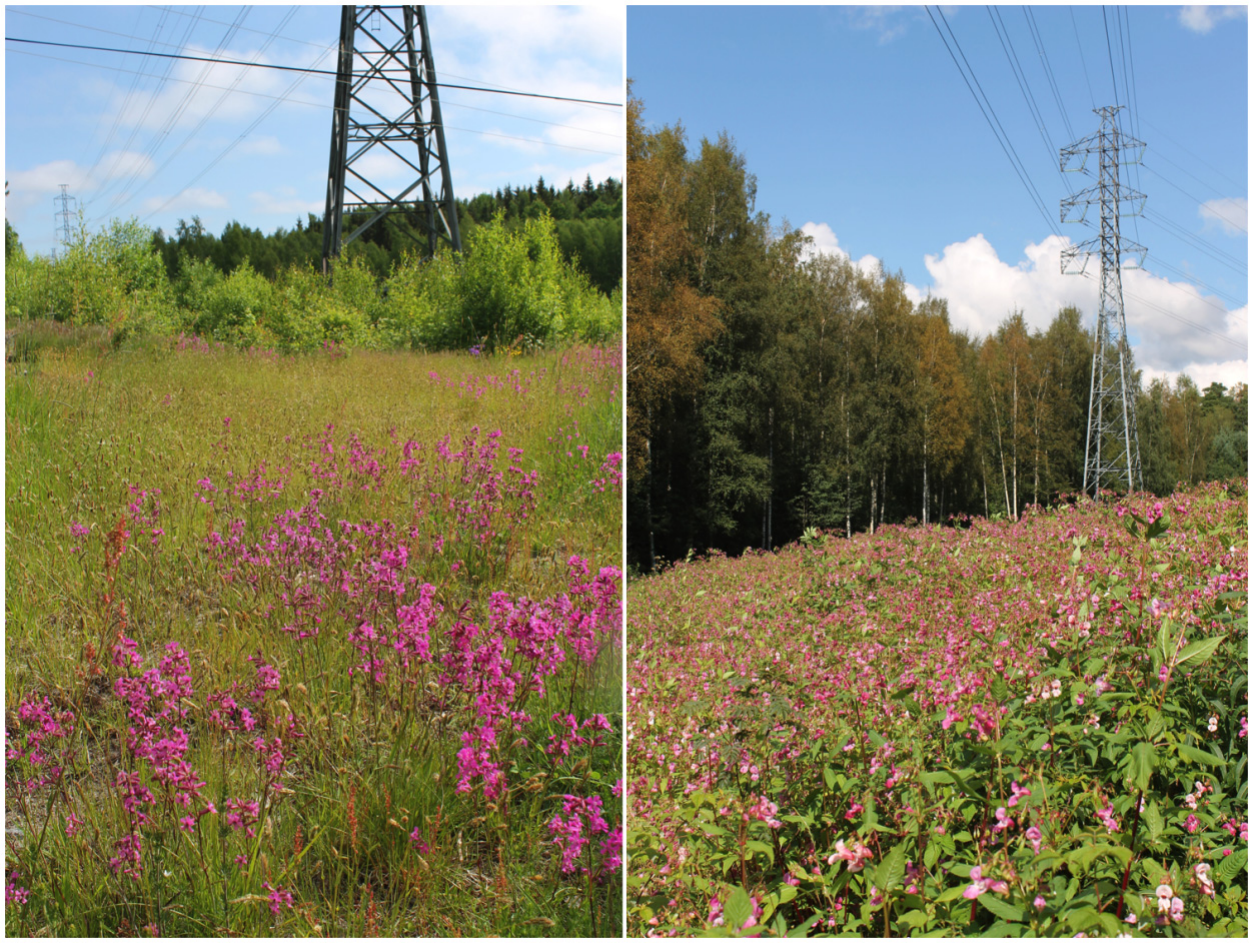
Two types of plant communities in power line corridors in Turku, Finland. On the left a corridor dominated by a species-rich dry grassland, with abundant *Viscaria vulgaris* Bernh. On the right, a corridor dominated by alien species, in this case *Impatiens glandulifera* Royle.

406 vascular plant taxa were encountered in the study corridors, with an average of 61.5 species per plot (Table 2, S1 and S2 Tables). 150 species (37% of all species) were classified as grassland species [29], with the majority being common grassland species typical to many early-successional habitats, such as *Anthoxanthum odoratum* L. and *Galium boreale* L. However, 42 grassland indicator species, such as *Helictotrichon pratensis* (L.) Besser and *Primula veris* L., were also encountered. The cover of both types of grassland species was generally low, with species often growing only as a few individuals per plot and gaining cover class values between 1 and 4. However, on some locations grassland species formed distinct patches of vegetation reminiscent of grasslands on traditional rural biotopes (Fig 2).

**Table 2:** Summary statistics of the species encountered on the study corridors.

	Total number (%)	Average (st dev)	Max	Min	Median
**Total species**	406 (100%)	61.5 (16.51)	92	26	62.5
**Grassland species:**					
Common grassland species	108 (26.6%)	25.8 (9.08)	46	7	26.5
Grassland indicator species	42 (10.3%)	2.94 (3.01)	12	0	2
**Alien species:**					
Indifferent alien species	72 (17.7%)	2.28 (3.04)	15	0	1
Invasive alien species	22 (5.4%)	2.3 (1.72)	7	0	2

94 alien species (23% of all species) were encountered in the study corridors, a substantial proportion compared to previous results in less urbanized corridors, such as 14% [6] and 9% [15]. In this study, the encountered species included ephemeral and indifferent aliens in the area, such as *Geranium macrorrhizum* L. [27], but also widespread, aggressive invaders, such as *Impatiens glandulifera* Royle and *Heracleum mantegazzianum* Sommier & Levier [30]. As with grassland species, the cover of both types of alien species ranged from a few individuals per plot to dominant status (Fig 2).

### Determinants for species number: Grassland species

Generalized linear models identified four statistically significant predictors for the number of common grassland species and six for the number of grassland indicator species in the study corridors, out of 20 initial independent predictors (Table 3). The remaining significant predictors for common grassland species were historical land covers of pastures and grassland, soil productivity and slope steepness. The significant predictors for grassland indicator species were historical land cover of pastures, soil moisture, corridor age, slope steepness, time since management and cover of surrounding forest. The effect of most of these was positive, but for example soil moisture had a negative effect on the number of grassland indicator species (Table 3).

**Table 3:** The final models for common grassland and grassland indicator species number in the study corridors. *q*-values indicate FDR-corrected *p*-values [4 1].

**Response:**	Number of common grassland species					
	**Predictors**	**Estimate**	**Std. Error**	**z value**	**Pr (>|z|)**	***q*-value**
	Intercept	3.27	0.023	139.987	< 0.001	
	History: Open areas and pasture	0.106	0.023	4.639	< 0.001	< 0.001
	History: Grassland	- 0.123	0.029	- 4.18	< 0.001	< 0.001
	Soil productivity	0.103	0.028	3.647	< 0.001	0.001
	Slope steepness	0.063	0.025	2.554	0.011	0.015
	Slope direction	0.042	0.026	1.585	0.113	0.113
	Shrub density	- 0.041	0.026	- 1.59	0.112	0.113
	**Null deviance / df.:**	234.80 / 70				
	**Residual deviance / df.:**	162.27 / 63				
	**AIC:**	537.88				
**Response:**	Number of grassland indicator species					
	**Predictors**	**Estimate**	**Std. Error**	**z value**	**Pr (>|z|)**	***q*-value**
	Intercept	1.208	0.069	17.479	< 0.001	
	History: Open areas and pasture	0.255	0.055	4.644	< 0.001	< 0.001
	Soil moisture	- 0.303	0.077	- 3.923	< 0.001	< 0.001
	Corridor age	0.242	0.074	3.254	0.001	0.003
	Slope steepness	0.169	0.068	2.504	0.012	0.03
	Time since management	0.203	0.092	2.197	0.028	0.05
	Cover of surrounding forests	- 0.141	0.066	- 2.142	0.032	0.05
	Cover of surrounding agricultural areas	- 0.148	0.077	- 1.925	0.054	0.07
	Shrub density	- 0.172	0.094	- 1.84	0.066	0.07
	Amount of debris	0.128	0.086	1.487	0.137	0.14
	**Null deviance / df.:**	140.151 / 70				
	**Residual deviance / df.:**	47.076 / 61				
	**AIC:**	281.36				

### Determinants for species number: Alien species

Generalized linear models identified nine statistically significant predictors for the number of indifferent alien species and three for the number of invasive alien species in the study corridors, out of 19 initial independent predictors (Table 4). The remaining predictors for indifferent alien species were soil productivity, light abundance, corridor width, the autocovariate, the covers of surrounding urban fabric, agricultural areas and forest as well as the historical land covers of cultivated field and grassland. Those for invasive alien species were the cover of surrounding urban fabric, soil productivity and time since management. Soil productivity had a positive effect on both species groups, while the cover of surrounding agricultural areas had a negative effect on indifferent alien species and time since management on invasive alien species.

**Table 4:** The final models for indifferent and invasive alien species number in the study corridors. *q*-values indicate FDR-corrected *p*-values [41].

**Response:**	Number of indifferent alien species					
	**Predictors:**	**Estimate**	**Std. Error**	**z value**	**Pr (>|z|)**	***q*-value**
	Intercept	1.004	0.077	13.014	< 0.001	
	Soil productivity	0.429	0.095	4.519	< 0.001	< 0.001
	Light abundance	0.399	0.098	4.07	< 0.001	< 0.001
	History: Grassland	- 0.219	0.085	- 2.587	0.01	0.029
	Corridor width	- 0.225	0.091	- 2.486	0.013	0.029
	Cover of surrounding urban fabric	0.21	0.104	2.016	0.044	0.044
	Cover of surrounding agricultural areas	- 0.236	0.117	- 2.013	0.044	0.044
	Cover of surrounding forests	0.237	0.11	2.159	0.031	0.044
	History: Cultivated field	- 0.178	0.086	- 2.079	0.038	0.044
	Autocovariate	0.18	0.089	2.027	0.043	0.044
	**Null deviance / df.:**	149.15 / 70				
	**Residual deviance / df.:**	62.96 / 61				
	**AIC:**	282.18				
**Response:**	Number of invasive alien species					
	**Predictors:**	**Estimate**	**Std. Error**	**z value**	**Pr (>|z|)**	***q*-value**
	Intercept	1.127	0.07	16.198	< 0.001	
	Cover of surrounding urban fabric	0.229	0.072	3.185	0.001	0.006
	Soil productivity	0.214	0.077	2.765	0.006	0.011
	Time since management	- 0.168	0.083	- 2.017	0.044	0.058
	Corridor width	0.144	0.088	1.639	0.101	0.101
	**Null deviance / df.:**	60.776 / 70				
	**Residual deviance / df.:**	29.792 / 66				
	**AIC:**	249.72				

### Determinants for species composition

**Fig 3:**
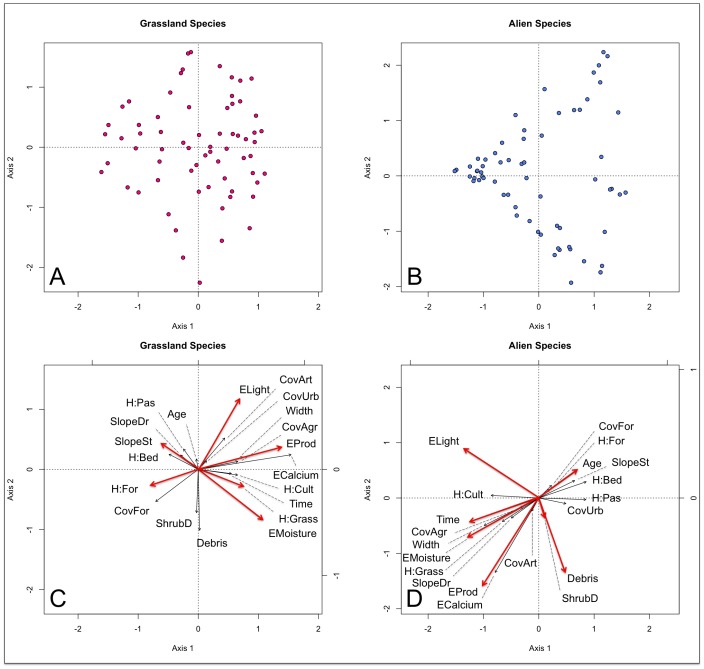
Distance-based redundancy analysis (Bray-Curtis distance) ordination on grassland and alien species on 67 plots in electric power line corridors. Panels A and B show only the plots and panels C and D the 20 environmental features used as explanatory variables. Red arrows indicate statistically significant variables after 500 permutations. For abbreviations, see Table 1.

In distance-based redundancy analysis the entire environmental data explained 52% of the variation in grassland species composition and 42% in alien species composition in the study corridors (Table 5). Permutation tests reduced the number of variables in the model to six (for grassland species) and seven (for alien species) (Table 6, Fig 3.). When only these variables were included in the model, the proportion of explained variance dropped to 36% and 25%, respectively (Table 5). In the ordination graph the species groups formed slightly different patterns concerning the environmental constraints, with plots presenting more clustering according to alien species composition (Fig 3).

**Table 5:** The proportion of constrained and unconstrained variation in the composition of grassland and alien species (expressed as Bray-Curtis distance), with all variables included and only significant variables included.

	Grassland species		Alien species	
	all variables	significant variables	all variables	significant variables
Total	1	1	1	1
Constrained	0.52	0.36	0.42	0.25
Unconstrained	0.48	0.64	0.58	0.75

**Table 6:** The models for grassland and alien species composition (expressed as Bray-Curtis distance), with only significant variables included.

**Response variable:**	Grassland species composition (Bray-Curtis distance)				
	**Predictors**	**df.**	**AIC**	**F**	**Pr (>F)**
	Slope steepness	1	179.24	2.22	0.020
	History: Grassland	1	179.41	2.39	0.010
	History: Forest	1	179.84	2.78	0.005
	Soil moisture	1	182.87	5.69	0.005
	Light abundance	1	183.62	6.42	0.005
	Soil productivity	1	185.12	7.93	0.005
**Response variable:**	Alien species composition (Bray-Curtis distance)				
	**Predictors**	**df.**	**AIC**	**F**	**Pr (>F)**
	Shrub density	1	220.72	1.67	0.035
	Corridor age	1	221.08	2.00	0.010
	Time since clear-cut	1	221.68	2.55	0.005
	Amount of debris	1	221.87	2.73	0.005
	Light abundance	1	221.9	2.75	0.005
	Soil moisture	1	222.03	2.87	0.005
	Soil productivity	1	222.88	3.66	0.005

When analyzed individually, the environmental variables explaining the largest amount of variation were soil calcium concentration (for grassland species) and soil productivity (for alien species) (Table 7). However, if a variable explained a large amount of variance in grassland species composition, it was likely to do the same with alien species, and vice versa. Indeed, Spearman correlation between the amount of variation the variables could explain in the two study groups was 0.541 (*p*-value 0.015).

**Table 7:** Environmental variables ranked according to the proportion of explained variation in the composition of grassland and alien species, when each variable was analyzed individually with the respective species data.

Grassland species composition		Alien species composition	
Environmental variable	Proportion of explained variance	Environmental variable	Proportion of explained variance
Soil calcium concentration	0.152	Soil productivity	0.062
Soil productivity	0.127	Light abundance	0.054
Soil moisture	0.100	Soil calcium concentration	0.051
Light abundance	0.079	Soil moisture	0.051
History: Forest	0.063	Amount of debris	0.045
History: Grassland	0.054	Time since clear-cut	0.043
Cover of surrounding forests	0.048	Corridor width	0.035
Corridor width	0.046	Corridor age	0.030
Slope steepness	0.045	Cover of surrounding urban fabric	0.028
Amount of debris	0.043	History: Open areas and pasture	0.026
Time since clear-cut	0.039	History: Cultivated field	0.025
Cover of surrounding agricultural areas	0.038	History: Forest	0.022
History: Cultivated field	0.036	Cover of surrounding artificial surfaces	0.020
History: Bedrock	0.031	History: Grassland	0.018
Cover of surrounding artificial surfaces	0.029	History: Bedrock	0.018
History: Open areas and pasture	0.028	Slope steepness	0.018
Corridor age	0.027	Cover of surrounding forests	0.016
Shrub density	0.026	Cover of surrounding agricultural areas	0.015
Slope direction	0.019	Slope direction	0.015
Cover of surrounding urban fabric	0.016	Shrub density	0.011

## Discussion

The main result of this study is that grassland and alien plant species may favor power line corridors with somewhat different characteristics, but the effects of individual environmental factors on the species composition in the two groups are correlated.

### Grassland species on power line corridors

According to this study, urban power line corridors most suitable to common grassland species are characterized by a history of pastures and productive soils on steep slopes, while corridors most suitable to grassland indicator species are old and likewise characterized by a history of pastures and steep slopes. Unsuitable corridors to common grassland species cross areas with a history as grassland, while those unsuitable to grassland indicator species cross mesic soils in densely forested areas.

Historical land use affects present vegetation patterns [48], and signs of agricultural practices can be evident in a plant community decades afterwards [49, 50]. We observed that power line corridors with a land cover history of pastures support large numbers of both common grassland species and grassland indicator species. This confirms earlier findings that grassland species may persist for decades after traditional management has ceased [21], and that even habitats that do not resemble traditional grasslands may support grassland species for extended periods of time [50]. The result that a history of actual grasslands was negatively correlated with the amount of common grassland species in power line corridors appears to contradict the previous, but can be explained by agricultural changes during the last century: Unlike the rocky, dry open areas and pastures unsuitable for cultivation, the vast majority of grasslands in southern Finland were transformed into cultivated and fertilized fields in late 19^th^ and early 20^th^ centuries [51, 8], and as such provide poor conditions for grassland species due to historical ploughing and fertilizing.

Steep topography and associated high levels of solar radiation promote high species richness on semi-natural grasslands [22, 52]. In addition, many grassland plants favor south-facing slopes with warm microclimates on the northern limit of their distribution [21]. The result that both common grassland and grassland indicator species favor power line corridors with steep slopes thus supports previous knowledge. In turn, the long management history of old corridors may explain why grassland indicator species increase in number with corridor age, as decades of regular management have been shown to produce open and low-growing plant communities in power line corridors [1].

High productivity coupled with frequent disturbance can result in high species diversity, as disturbance prevents the competitive exclusion of species [53]. This may explain why the number of common grassland species was positively correlated with soil productivity: Corridor management acts as sufficient disturbance that enables a large amount of species to coexist on productive corridors that would otherwise be dominated by fewer, competitive species [2, 3]. However, soil productivity was highly correlated with soil calcium concentration (S3 Table), although the latter was omitted from the final models during variable selection. Correlation between the two indicator values has been observed previously [54], and is caused by soil acidity affecting nitrogen mineralization rates, thus contributing to soil productivity [55].

The result that grassland indicators decrease in number with increasing soil moisture is likely a spurious correlation caused by the species’ preference for steep and therefore also relatively dry slopes [22, 52]. Increasing shrub density also showed a negative, although non-significant correlation with both species groups, and the results are likely interrelated: Corridors with mesic soils are quickly covered by shrubs and tree saplings [4], rendering them unsuitable for grassland plants adapted to open environments and abundant light [56]. This also explains why an increase in surrounding forest cover correlated with decreasing number of grassland indicator species, as corridors crossing densely forested areas are easily covered by forest floor shrubs and saplings.

### Alien species on power line corridors

According to this study, urban power line corridors favored by indifferent alien species cross areas close to both forests and the urban fabric, and are characterized by productive soils and abundant light. Corridors favored by invasive alien species likewise cross areas close to the urban fabric on productive soils. Unsuitable corridors for invasive species have been clear-cut several years ago, while corridors crossing agricultural areas or those with a history as cultivated fields appear unsuitable for only indifferent alien species.

Recent disturbance, a surplus of unused resources and propagule pressure increase the likelihood of invasion in communities [57, 16], and both indifferent and invasive alien species followed these predictions in the studied corridors. By producing disturbance, corridor management increases the availability of light and other resources on the corridor, while increasing time since previous clear-cut decreases them due to natural succession. Propagule pressure, on the other hand, is heaviest on corridors in densely urbanized areas, due to the disturbed nature of urban land use types as well as the deliberate import of e.g. horticultural species [20].

The amount of both types of alien species also had a positive correlation with soil productivity. Although no general relationship between productivity and community invasion should exist [16], experimental studies have shown that frequent disturbance coupled with high fertility increases the likelihood of invasion [57].

Indifferent alien species had a tendency to be found in densely forested areas, but to avoid both agricultural areas and those with a history as cultivated fields or grasslands. This partly contradicts previous research, as mature forests present only low levels of alien invasion in Europe, while agricultural landscapes appear highly susceptible to invasion due to associated disturbance and structural heterogeneity [58]. The result may be related to the partial redundancy of present day land cover data, and should be investigated further with a more robust set of data. The amount of indifferent alien species also showed a significant, although small level of spatial structuring. This may be caused by either the environmental conditions favoring these species being spatially structured, or by the limited dispersal of the species along the corridors [39].

### Determinants for species composition

The most important environmental factors controlling both grassland and alien species composition included soil calcium concentration, light abundance, soil moisture and soil productivity. Their contribution to species composition in both groups is expected, as they are key factors in shaping plant communities and describe basic resources every plant needs to survive [52]. In addition, slope steepness and a history of both grasslands and forests were statistically significant predictors for grassland species composition, while corridor age, shrub density and amount of debris for that of alien species composition.

Slope steepness can drive species turn over in grassland communities, as it reflects a gradient of solar radiation, temperature and moisture [52]. Historical land cover, in turn, can influence species turn over by e.g. creating conditions which some species can´t tolerate and others favor. As for alien species, shrub density and amount of debris both reflect a gradient of progressing succession. This gradient drives species turn over in alien communities, as some aliens are likely capable of invading only the most open corridors, while some others can tolerate corridors with more shrubs.

Individual environmental variables were found to explain similar proportions of variation in the composition of the two plant groups. This likely indicates that power line corridors are suitable environments for the two groups for similar reasons: Both require regular disturbances and the resulting early-successional conditions to survive on the corridors. Grassland species require them because they are adapted to open environments with low levels of competition [59]. In turn, alien species require them because the invasibility of a community demands disturbance and the related release of resources and relaxed competition [16].

### Implications for conservation and management

According to this study, including power line corridors in grassland conservation will be most successful in old, dry corridors with steep slopes and a history of use as pastures. Such corridors can be especially important habitats for grassland plants in areas where grazing or other traditional practices have ceased and difficult to re-establish, such as urban areas. In turn, deterring the spread of alien species in power line corridors is especially needed in recently clear cut corridors on productive soils and close to the urban fabric. Utilizing such power line corridors in the struggle against invasive alien species is especially important in urban areas, where invasive species may already subject native biotas to heavy competition.

In conclusion, urban power line corridors can at best contribute to biodiversity conservation by providing habitats for native grassland species. At worst they can provide further footholds for invasive alien species, which already pose problems in urban areas. Ecologically informed management considering both safe electricity transportation and nature conservation could promote the former and prevent the latter.

## Supporting Information

S1 Table Data on vascular plant species on 71 plots on electric power line corridors in Turku, SW Finland. (CSV)

S2 Table Data on environmental conditions on 71 plots on electric power line corridors in Turku, SW Finland. (CSV)

S3 Table Pearson correlation matrices between the environmental variables used in the generalized linear models. (CSV)

S1 Text Descriptions for the species and environmental data. (DOCX)
