# Supplementary material for: Urban Power Line Corridors as Novel Habitats for Grassland and Alien Plant Species in South-Western Finland
Source: PLoS One. 2015 Nov 13;10(11):e0142236. doi: 10.1371/journal.pone.0142236 (PMC4643934; doi:10.1371/journal.pone.0142236)
Supplement: S1 Text — (DOCX) [file pone.0142236.s004.docx]

**Supporting information metadata**

**S1 Table:**

**Scientific name**: Name of observed species [26, 27].

**Conservation status**: The IUCN conservation status of each species according to national [10] and regional* estimates. RT = regionally threatened, NT = near threatened, VU = vulnerable.

**Grassland species**: Indicates if the observed species can be classified as either a common grassland species or a grassland indicator species [29].

**Alien species**: Indicates if the observed species can be classified as either an indifferent alien species or an invasive alien species [26, 30].

**Ellenb. light, Ellenb. moisture, Ellenb. reaction, Ellenb. nitrogen**: The indicator values for light, moisture conditions, soil reaction (in the article soil calcium concentrations) and soil nitrogen (in the article soil productivity) [33, 34] for each observed species, when available. Lacking values are indicated by -.

**Species coverage on plots**: The cover of each species on each plot, evaluated with 9 category values: 0 = species not present, 1 = < 0.125 % coverage, 2 = 0.125-0.5 %, 3 = 0.5-2 %, 4 = 2-4 %, 5 = 4-8 %, 6 = 8-16 %, 7 = 16-32 %, 8 = 32-64 % and 9 = > 64 % coverage. Adapted from [28].

***** Ryttäri T, Kalliovirta M, Lampinen R. Suomen uhanalaiset kasvit. Helsinki: Tammi; 2012. Finnish.

**S2 Table:**

**North and East coordinate (ETRS-TM35FIN):** Coordinates of center of the study plot.

**Corridor age:** Time in years since the corridor was established.

**Slope steepness and direction:** Categorical variables describing the angle and direction of slope on the plot.

**Corridor width:** Categorical variable describing the corridor width in meters.

**Time since clear-cut:** Time in years since the corridor was clear-cut previously.

**Amount of debris:** Categorical variable describing the amount of woody debris on the plot produced by clear-cuts and left on the corridor ground.

**Shrub density:** Categorical variable describing the amount of shrubs and saplings growing on the plot.

**Light abundance, soil moisture, soil calcium and soil productivity:** Plot-wise mean Ellenberg indicator values [33, 34] based on the species-specific values of the species found on each plot.

N.b.: Plot-wise values presented here are based on all species observed on the plots. In the analyses determining the amount and composition of grassland or alien plants, the species in question and their species-specific indicator values were omitted from the plot-wise mean values, in order to avoid circular reasoning.

**Autocovariate_commongrass, Autocovariate_indicatorgrass, Autocovariate_indifferentalien and Autocovariate_invasivealien:** Values describing spatial structure in the number of species in each group. Calculated with the autocov_dist -function in the spdep -package of R [45].

**Cover of surrounding urban fabric, artificial surfaces, agricultural areas and forests:** The coverage of each present day CORINE land cover class in a circle 100 m in diameter surrounding the plot [32].

**History: Bedrock, Open areas and pasture, Grassland, Cultivated field and Forest:** Categorical variables describing the prevalent land cover on plot location in the late 19^th^ century [31]

**Number of all species, common grassland species, grassland indicator species, indifferent alien species and invasive alien species:** The amount of species on each plot that could be classified [29, 26, 30] into any of said groups.

**S3 Table:**

Pearson -correlation matrices for the environmental variables used in each generalized linear model.
